# Updating the “Meswa Bridge” Locality Name to “Tonde Bridge”

**DOI:** 10.1002/ajpa.70166

**Published:** 2025-11-14

**Authors:** Joshua Siembo, Cliff Ochieng Odida, Samuel N. Muteti, Kieran P. McNulty

**Affiliations:** ^1^ Rusinga Island Prehistory Organization Mbita Kenya; ^2^ Palaeontology Section National Museums of Kenya Nairobi Kenya; ^3^ Department of Ecology, Evolution, & Behavior University of Minnesota St. Paul Minnesota USA

The Early Miocene locality called Meswa Bridge (0°08′10.2″ S, 35°12′20.9″ E), located near Muhoroni town in Kisumu County, Kenya, represents a small but important fossil deposit. Inferred to be close in age to the Oligocene‐Miocene boundary, and as the type locality for the ape species *Proconsul meswae* (Harrison and Andrews 2009), Meswa Bridge has particular importance for understanding the early evolution of Hominoidea. In the course of outreach efforts in this region, we discovered that the name “Meswa Bridge” is not recognized by the people who currently live near the fossil site. At their request, and to better represent the communities in which fossil excavations have taken place, we announce here a formal change of the name of this fossil locality to “Tonde Bridge.” Similar to previous locality name changes in Kenya (East Rudolf; West Stephanie), the National Museums of Kenya accession code (KNM‐ME) will remain the same. Likewise, and at the stated preference of community leaders, this change would not impact taxonomic nomenclature that references the previous locality name (e.g., *Proconsul meswae*).

We first became aware of the name discrepancy when working in the Koru region as part of the REACHE (Research on Eastern African Catarrhine and Hominoid Evolution) paleontological team. We inquired with people from that area about the location of “Meswa Bridge” and were led to a river crossing up in the hills. This, according to some Koru residents, was the Meswa River and hence was “Meswa Bridge.” However, we were nowhere near the place where Pickford (1986) mapped the Meswa Bridge fossil locality—on the rift valley floor near Muhoroni—and, indeed, a short survey of the area returned no fossil remains.

We did relocate the original fossil locality, and although no fossil remains were found at that time, Pickford's (1986, Fig. II‐4: 27) coordinates and detailed map of the locality confirmed the location. In fact, the team did not expect to find fossils at Meswa Bridge, given the limited scope of fossiliferous deposits reported there (Andrews et al. 1981) and the history of excavation (see Harrison and Andrews 2009). Regardless, it was clear that local geographic names were inconsistent with names used by western researchers.

Recent outreach in that area by two of us (JS, COO) further clarified how the local community perceives the identity of the river and of the fossil outcrop. In cooperation with the Assistant County Commissioner of Muhoroni Division, the Chief of Tonde Location, and the Assistant Chief of the Tonde Sub‐location, we convened a *baraza* (meeting of different groups) to share with the community our knowledge of the ancient animals that once inhabited their land. During this meeting, with approximately 400 people in attendance, there was widespread confusion expressed about the fossil site being incorrectly named. Although that community could not identify precisely where the name “Meswa” came from, the word itself is of Kalenjin origin. Binge (1962) labeled the river “Meswa” in the geological map accompanying his *Report*, and we hypothesize that western scientists working for the geological survey likely obtained that name near the river's origin in the hills, carrying it with them even as the river changed names according to local geography and culture.

Local history does not resolve a single name for the river alongside the fossil locality or the bridge that spans it. There is a second drainage only half a kilometer north, and these merge about half a kilometer southwest of the site (Figure 1). As these streams flow across the land and across history, they have picked up multiple names. Community members variously refer to the river as “Tonde” or “Ogwande,” with the latter name identified by village elders as an older one. The name “Kipturi” or “Kipturu,” identified by Pickford (1986, Fig. II‐4: 27) as an alternate name for the river, originates further upstream near the junction of Kisumu, Kericho, and Nandi County borders (Figure 1). From this variety of historic designations, there may not have been an obvious local choice for the site name at the time of the original fossil excavation, whereas Binge's (1962) geological survey of the area clearly marked the adjacent drainage as River Meswa (Figure 1). Importantly, however, the area is now a formal municipal entity identified by the Kenyan government, with only one of those historic names attached to the land and river. The community members gathered at the *baraza* were thus unanimous in (1) situating the fossil locality within Tonde village, (2) identifying the nearby river crossing as Tonde Bridge, and (3) naming the drainage adjacent to the fossil locality as River Tonde: A name derived from indigenous ropes or vines (*tonde* in the Dholuo language) found hanging in the gallery forest that once lined the river.

**FIGURE 1:**
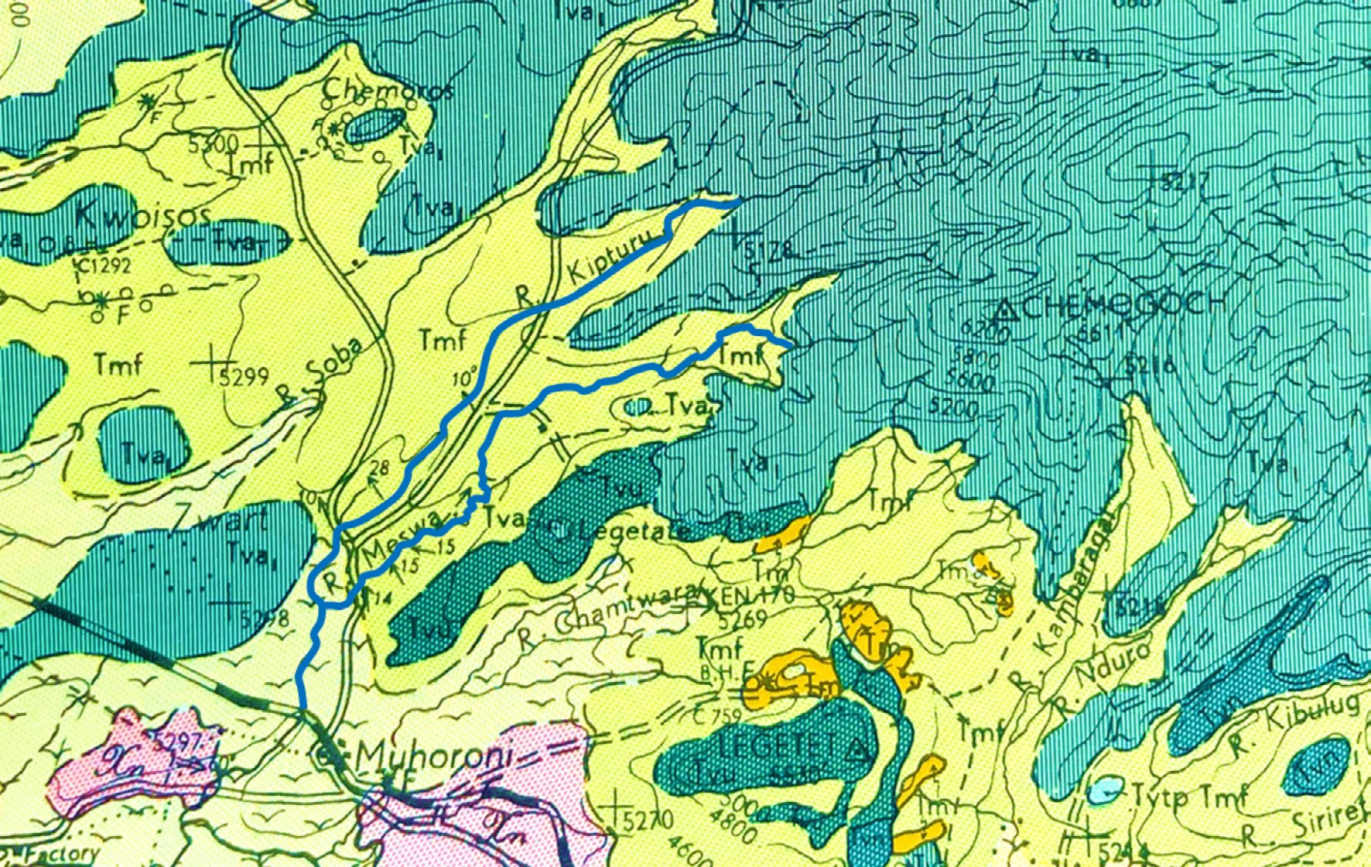
Excerpt from “Geological Map of the Kericho Area,” published with Report #50 from the Geological Survey of Kenya (Binge 1962), showing the region north of Muhoroni town, with the Kipturu (north) and Meswa (south) drainages highlighted with blue lines. The fossil locality is located on this map just after the base of the letter “s” in “Meswa.” The bridge itself is southwest of that, where the river crosses the double black line that indicates the roadway. A lower‐resolution version of this map can be accessed online at https://edepot.wur.nl/484768 [accessed October 16, 2025]. Photo courtesy of Z. Sims.

Based on the concerns of the villagers regarding the river's identity, the Assistant Chief of Tonde Sub‐location, Mr. Joseph Oyuga, and his committee formally requested us to take this issue to the National Museums of Kenya and to the scientific community. In response to our written report, the Director of Antiquities, Sites and Monuments and the Head of the Earth Science Department at the National Museums of Kenya agreed to grant the request of the Tonde community and change the locality name to “Tonde Bridge.” We submit this announcement as well to the community of biological anthropologists so that the correct name can be used in future excavations and publications.

## Author Contributions

**Joshua Siembo:** conceptualization (equal), project administration (equal), supervision (lead), writing – original draft (equal), writing – review and editing (supporting). **Cliff Ochieng Odida:** conceptualization (equal), project administration (equal), supervision (equal), writing – review and editing (supporting). **Samuel N. Muteti:** resources (supporting), writing – review and editing (supporting). **Kieran P. McNulty:** conceptualization (supporting), funding acquisition (lead), project administration (supporting), resources (lead), supervision (supporting), writing – original draft (equal), writing – review and editing (lead).

## Conflicts of Interest

The authors declare no conflicts of interest.

## Data Availability

There were no data used in this manuscript.
